# Validation and analysis of prognostic scoring systems for critically ill patients with cirrhosis admitted to ICU

**DOI:** 10.1186/s13054-015-1070-y

**Published:** 2015-10-13

**Authors:** Joseph Campbell, Joanne McPeake, Martin Shaw, Alex Puxty, Ewan Forrest, Charlotte Soulsby, Philp Emerson, Sam J. Thomson, Tony M. Rahman, Tara Quasim, John Kinsella

**Affiliations:** Academic Unit of Anaesthesia, Pain and Critical Care, University of Glasgow, Room 2.73, Level 2, New Lister Building, Glasgow Royal Infirmary, 10-16 Alexandra Parade, Glasgow, G31 2ER UK; Medical Physics, NHS Greater Glasgow and Clyde, Level 2, New Lister Building, Glasgow Royal Infirmary, 10-16 Alexandra Parade, Glasgow, G31 2ER UK; Intensive Care Unit, NHS Greater Glasgow and Clyde, 84 Castle Street, Glasgow, G4 OSF UK; Department of Gastroenterology, NHS Greater Glasgow and Clyde, 84 Castle Street, Glasgow, G4 OSF UK; Clinical lead for Gastroenterology & Hepatology (Worthing), Western Sussex Hospitals NHS Foundation Trust, Worthing, BN11 2DH UK; Department of Gastroenterology & Hepatology, The Prince Charles Hospital, Brisbane, Queensland Australia; College of Medicine & Dentistry, James Cook University, Cairns, Queensland Australia

## Abstract

**Introduction:**

The number of patients admitted to ICU who have liver cirrhosis is rising. Current prognostic scoring tools to predict ICU mortality have performed poorly in this group. In previous research from a single centre, a novel scoring tool which modifies the Child-Turcotte Pugh score by adding Lactate concentration, the CTP + L score, is strongly associated with mortality. This study aims to validate the use of the CTP + L scoring tool for predicting ICU mortality in patients admitted to a general ICU with cirrhosis, and to determine significant predictive factors for mortality with this group of patients. This study will also explore the use of the Royal Free Hospital (RFH) score in this cohort.

**Methods:**

A total of 84 patients admitted to the Glasgow Royal Infirmary ICU between June 2012 and Dec 2013 with cirrhosis were included. An additional cohort of 115 patients was obtained from two ICUs in London (St George’s and St Thomas’) collected between October 2007 and July 2009. Liver specific and general ICU scoring tools were calculated for both cohorts, and compared using area under the receiver operating characteristic (ROC) curves. Independent predictors of ICU mortality were identified by univariate analysis. Multivariate analysis was utilised to determine the most predictive factors affecting mortality within these patient groups.

**Results:**

Within the Glasgow cohort, independent predictors of ICU mortality were identified as Lactate (p < 0.001), Bilirubin (p = 0.0048), PaO_2_/FiO_2_ Ratio (p = 0.032) and PT ratio (p = 0.012). Within the London cohort, independent predictors of ICU mortality were Lactate (p < 0.001), PT ratio (p < 0.001), Bilirubin (p = 0.027), PaO_2_/FiO_2_ Ratio (p = 0.0011) and Ascites (p = 0.023). The CTP + L and RFH scoring tools had the highest ROC value in both cohorts examined.

**Conclusion:**

The CTP + L and RFH scoring tool are validated prognostic scoring tools for predicting ICU mortality in patients admitted to a general ICU with cirrhosis.

## Introduction

The prevalence of liver disease in Scotland has been increasing over the last 30 years. Mortality due to liver disease is one of the few causes of death that is increasing [1]. There is a similar picture in England and Wales, with liver disease being the fifth most common cause of mortality after heart disease, cancer, stroke, and respiratory disease. This is in contrast to most Western European countries, which have seen a decline [1, 2].

Liver disease accounts for an increasing proportion of Intensive Care Unit (ICU) and hospital admissions. Admissions rose by 71 % in male patients and 43 % in female patients between 1990 and 2003. This change has been mainly attributed to alcohol, which accounted for 85 % of liver disease deaths in 2007 [3]. Overall, patients with cirrhosis account for 15 % of Glasgow Royal Infirmary ICU admissions, and 3.3 % of ICU admissions in St George’s and St Thomas’ Hospitals. Patients with liver disease admitted to ICU have poor outcomes and a complex disease process. Mortality in these patients is widely documented in literature, with a meta-analysis of seventeen papers in 2010 showing the weighted mean ICU and hospital mortality to be 45 % and 58 %, respectively [4, 5].

There are currently no validated prognostic scoring tools to predict ICU outcome in patients with cirrhotic liver disease within the general ICU setting that can be calculated quickly at a patient’s bedside. Existing hepatic scoring tools are designed for a specific use, for example, the Child-Turcotte Pugh (CTP) score was designed to predict mortality following surgical treatment of oesophageal varices, and the United Kingdom model for end-stage liver disease (UKELD) was designed to assess patients for transplant in the UK [6, 7].

In research conducted at Glasgow Royal Infirmary, existing scoring tools (the CTP, UKELD, model of end-stage liver disease (MELD), Glasgow alcoholic hepatitis score (GAHS), sequential organ failure assessment (SOFA), the acute physiology and chronic health evaluation II (APACHE II), and the chronic liver failure-sequential organ failure assessment score (CLIF-SOFA)) did not reach the level of clinical usefulness based on receiver operating characteristic (ROC) curve analysis of an area under the curve (AUC) of ≥0.8 [8-10]. Therefore, these existing tools may not be clinically useful for predicting ICU mortality. Analysis of this cohort found lactate, bilirubin, ascites, and prothrombin time (PT) ratio as independent predictors of outcome [11]. Other published studies have shown promising results for predicting outcome in patients with cirrhosis using the SOFA and MELD scoring tools, and have also demonstrated that the current CTP score is not the most effective tool for predicting outcome in patients with cirrhosis [12, 13].

The relationship between blood lactate concentration on admission to the ICU and mortality in patients with cirrhosis is widely demonstrated within the literature [14-17]. Despite this, only one existing liver-specific scoring tool, the Royal Free Hospital score (RFH) includes lactate, which is validated for use in a tertiary hepatic treatment centre [18]. As a result, in a previous study [11] lactate was incorporated into an existing scoring tool, the CTP, to produce two novel tools. The CTP score was chosen due to its categorical variables that can easily be calculated at a patient’s bedside. One scoring tool (CTP-L) splits the lactate into bands and awards 1, 2 or 3 points, which is similar to the other variables in the CTP score. The other tool (CTP + L) adds the raw value of lactate (mmol/L) onto the existing CTP score. These unvalidated tools performed well in a single cohort of patients, but results need to be validated in patients from another centre to demonstrate the usefulness of the scoring tool [11]. This study aims to validate these newly created scoring tools as prognostic measures of ICU outcome using data obtained from another ICU centre, and to determine the most predictive factors for predicting ICU outcome within these cohorts.

## Methods

Data collection for the previous study took place between June 2012 and May 2013, and 59 patients were recruited from the Glasgow Royal Infirmary (GRI) ICU. This is a 20-bed facility with a large gastroenterology unit, but is not a tertiary hepatic transplant centre [11]. An additional cohort of 25 patients from the GRI was recruited by extension of the data collection period by 6 months, giving a combined total of 84 patients. Data were collected as part of routine data collection within the department and no additional consent was required. Ethics approval was granted by the Local Research Ethics Committee (West of Scotland Research Ethics Committee, approved 20 March 2012, REC reference; 12/WS/0039, Chair; Dr Gregory Ofili) for the original data collection and the extension of data collection.

Inclusion was based on the presence of cirrhosis on admission to the ICU in any patient over 18 years old. Cirrhosis was diagnosed either histologically following biopsy or clinically by evidence of portal hypertension and one of the following: ascites, encephalopathy or oesophageal varices. Diagnosis was confirmed by an independent clinician.

Clinical and demographic data were obtained from patients’ electronic records (CareVue, Philips IntelliVue Clinical Information Portfolio (ICIP) Revision D.03, Warrick, Naik, Avis, Fletcher, Franklin, Inwald 2011) and WardWatcher (Critical Care Audit Limited, Yorkshire). These are validated and complete clinical information systems [19]. First available clinical test results after admission to the ICU were recorded and used to calculate scores using all scoring tools. Clinical and biochemical data collected included sodium, potassium, urea, arterial lactate, creatinine, white cell count, bilirubin, PT ratio, albumin, platelets, arterial partial pressure of oxygen (PaO_2_), arterial partial pressure of carbon dioxide (PaCO_2_), PaO_2_/ inspired oxygen fraction (FiO_2_) ratio (calculated from the arterial gas sample), Glasgow coma scale (GCS), mean arterial blood pressure (MAP), noradrenaline dose, ascites and encephalopathy grade. Demographic information was also recorded relating to: age, gender, reason for admission, and Scottish Index of Multiple Deprivation (SIMD). The SIMD scores deprivation based on postcode and takes into account employment, income, health, education and crime, and is only applicable within Scotland [20]. The Indices of Deprivation is a deprivation score that is valid within England, but the required information was not available at the time of data collection so the score is not included [21]. West Haven criteria encephalopathy scores and ascites scores were collected pre-intubation in all patients.

A second previously published cohort of 115 ICU patients with cirrhosis was obtained from St Thomas’ Hospital and St George’s Hospital in London. Data were collected over a period of 20 months between 31 October 2007 and 1 July 2009. These patients were recruited for a demographic study in cirrhotic patients within a general ICU population. Scoring tools for these patients were recalculated based on raw data[5].

Both general ICU and liver-specific scoring tools were used. The general ICU scoring tools calculated were the APACHE II and the SOFA score [22, 23]. Liver-specific scoring tools used were the CTP, UKELD, MELD, CLIF-SOFA and the RFH score [6, 7, 18, 24, 25].

Of the two new scoring tools, CTP-L and CTP + L, the latter had achieved a higher AUC on ROC curve analysis in the cohort upon which it was designed. As a result, this was the only scoring tool used [11]. A breakdown for the calculation of the CTP + L score can be seen in Table 1. The GRI data are referred to as the Glasgow dataset, and the St Thomas’ and St George’s data are referred to as the London dataset.

**Table 1:** Calculation of the Child-Turcotte Pugh + lactate (CTP + L) score

Variable	1 Point	2 Points	3 Points
Bilirubin (μmol/L)	<34	34 50	>50
Albumin (g/L)	>35	28–35	<28
INR (or PT ratio)	<1.7	1.71–2.30	>2.3
Ascites grade	None	Mild	Severe
Encephalopathy grade	None	Grade I/II	Grade III/IV
Serum arterial lactate	Addition of value in mmol/L to score obtained from above categories		

1, 2 or 3 points are awarded for the five categories below, which form the CTP score. The serum arterial lactate concentration is added to the CTP score to form the CTP + L score. *INR* international normalized ratio, *PT* prothrombin time

Within the Glasgow data, encephalopathy scores were collected prospectively pre-intubation in all patients to record accurate values. In other studies, including the data from the London dataset, the encephalopathy score was presumed to be 2, as pre-intubation scores were not available [5]. To test if the collection of pre-intubation encephalopathy scores was necessary, the Glasgow dataset was modified to compare the CTP score with correct encephalopathy, with the CTP score excluding encephalopathy scores.

### Statistical analysis

Univariate analysis was performed to identify variables significantly related to ICU outcome. The Welch independent samples *t* test was performed for continuous parametric data, and the Mann-Whitney *U* test was used for non-parametric continuous data. Pearson’s Chi squared test with Yates’ continuity correction (where appropriate) or Fisher’s exact test for count data were used for categorical data. All assumptions for statistical tests were met and *p* <0.05 was considered statistically significant. All missing data were kept blank.

Scoring tools were applied to both datasets, and compared using the AUC and optimum cutoff point determined by the Youden’s index from ROC curves. Statistical models were produced by binary logistic regression for individual variables in both datasets against ICU mortality, with model selection based on analysis of variance (ANOVA) and Akaike information criterion (AIC) values. The optimum cut point from ROC curves produced for models was used to predict outcomes in the other dataset, and goodness-of-fit was compared using the Chi squared test and the phi coefficient. ROC curves were directly compared using DeLong’s test for correlated ROC curves and the Chi squared test. An independent statistician provided assistance with the analysis in this study. Statistical analysis was performed using RStudio version 0.98.493 (R Foundation for Statistical Computing: Vienna, Austria) [26-28].

## Results

There were 84 patients from the Glasgow dataset and 115 patients from the London dataset initially included in the analysis. During model selection, five patients from the Glasgow dataset and one patient from the London dataset were excluded from subsequent data analysis due to missing values, leaving 79 and 114 patients in each group, respectively.

Univariate analysis of the Glasgow dataset demonstrated that significant predictors of mortality were lactate (*p* <0.001), bilirubin (*p* = 0.0048)_,_ PaO_2_/FiO_2_ ratio (*p* = 0.032), PT ratio (*p* = 0.012). Mean age of patients in the Glasgow dataset was 50 years, and 66 % of patients were from the most deprived category of the SIMD. A summary of patient data collected and univariate analysis results can be seen in Table 2.

**Table 2:** Glasgow Royal Infirmary (GRI) dataset patient characteristics and univariate analysis

Variable	All patients (n = 84)	ICU survivor (n = 59)	ICU death (n = 25)	*P* value
Age mean (range)	50.2 (29–80)	49.7 (29–80)	51.4 (32–72)	0.55
Gender, male, n (%)	59 (70.2 %)	41 (69.5 %)	18 (72.0 %)	1.00
SIMD category				0.44
1–2 (deprived), n (%)	68 (81.0 %)	46 (78.0 %)	22 (88.0 %)	
3–5 (non-deprived), n (%)	16 (19.0 %)	13 (22.0 %)	3 (12.0 %)	
Alcoholic aetiology, n (%)	70 (83.3 %)	48 (81.3 %)	22 (88.0 %)	0.54
Encephalopathy, any, n (%)	29 (34.5 %)	19 (32.2 %)	10 (40.0 %)	0.66
Ascites, any, n (%)	35 (41.7 %)	22 (37.3 %)	13 (52.0 %)	0.31
Sodium, mmol/L, mean (range)	136.4 (113.0–151.0)	136.7 (113.0–151.0)	135.7 (128.0–147.0)	0.52
Potassium, mmol/L, mean (range)	4.1 (2.6–7.0)	4.0 (2.6–7.0)	4.3 (2.9–5.9)	0.27
Urea, μmol/L, median (IQR)	8.1 (4.1–12.7)	7.7 (4.4–12.1)	9.2 (4.0–14.5)	0.44
Lactate, mmol/L, median (IQR)	1.9 (1.3–2.7)	1.7 (1.2–2.2)	4.1 (2.0–8.0)	<0.001
Creatinine, μmol/L, median (IQR)	81.5 (57.8–158.8)	75.0 (57.5–138.5)	144.0 (69.0–199.0 )	0.056
WCC, × 10^9^/L, mean (range)	13.6 (0.8–41.7)	13.6 (0.8–36.4)	13.6 (1.5–41.7)	0.99
Bilirubin, μmol/L, median (IQR)	45.5 (22.3–106.8)	33.0 (18.0–76.5)	71.0 (40.0–182.0)	0.0048
PT ratio, median (IQR)	1.5 (1.2–2.0)	1.5 (1.2–1.8)	1.8 (1.5–2.5)	0.012
Albumin, g/L, mean (range)	21.8 (8.0–79.0)	22.6 (8.0–79.0)	19.8 (10.0–33.0)	0.14
Platelets, × 10^9^/L, mean (range)	138.5 (6.0–487.0)	145.4 (25.0–487.0)	122.2 (6.0–371.0)	0.31
PaO_2_, kPa, median (IQR)	12.4 (9.9–18.1)	12.5 (9.8–19.2)	12.1 (10.0–15.8)	0.65
PaO_2_/FiO_2_ ratio, median (IQR)	21.8 (12.8–35.6)	24.5 (14.8–38.6)	15.8 (11.7–25.3)	0.032
APACHE II, mean (range)	23.5 (2.0–47.0)	21.5 (2.0–39.0)	28.3 (14.0–47.0)	<0.001
SOFA, mean (range)	9.7 (3.0–20.0)	8.6 (3.0–15.0)	12.3 (4.0–20.0)	<0.001
MELD, median (IQR)	19.0 (13.3–25.0)	16.0 (12.0–21.0)	25.0 (20.0–30.0)	<0.001
UKELD, median (IQR)	52.0 (47.3–58.0)	51.0 (47.0–53.0)	58 (50.0–61.0)	0.0016
CTP, median (IQR)	9.0 (7.3–11.0)	9.0 (7.0–11.0)	11.0 (9.0–13.0)	0.012
CTP + L, median (IQR)	11.0 (9.0–14.3)	10.0 (9.0–12.5)	15.0 (13.0–19.0)	<0.001
CLIF-SOFA, median (IQR)	10.0 (7.0–12.3)	9.0 (6.0–11.0)	12.0 (10.0–14.0)	<0.001
RFH, median (IQR)	0.41 (–0.93–2.00)	−0.52 (−1.64–0.73)	2.12 (0.52–3.14)	<0.001

*SIMD* Scottish Index of Multiple Deprivation, *WCC* white cell count, *PT* prothrombin time, *PaO*_*2*_, arterial partial pressure of oxygen, *FiO*_*2*_, inspired oxygen fraction, *APACHE II* acute physiology and chronic health evaluation II, *SOFA* sequential organ failure assessment, *MELD* model of end-stage liver disease, *UKELD* United Kingdom model for end-stage liver disease, *CTP* Child-Turcotte Pugh, *CTP + L* Child-Turcotte Pugh + lactate, *CLIF-SOFA* chronic liver failure sequential organ failure assessment, *RFH* Royal Free Hospital score

Univariate analysis of the London dataset showed that significant predictors of mortality were PT ratio (*p* <0.001), lactate (*p* <0.001), PaO_2_/FiO_2_ ratio (*p* = 0.0011), bilirubin (*p* = 0.027), and the presence of ascites (*p* = 0.023). Mean age for patients in the London dataset was similar to the Glasgow dataset at 51 years. A summary of the London dataset patient data can be seen in Table 3.

**Table 3:** London Dataset patient characteristics and univariate analysis

Variable	All patients (n = 115)	ICU survivor (n = 72)	ICU death (n = 43)	*P* value
Age, mean (range)	50.9 (22.0–82.0)	50.0 (28.0–71.0)	52.44 (22.0–82.0)	0.30
Gender, male, n (%)	78 (67.8 %)	51 (70.8 %)	27 (62.8 %)	0.49
Ascites, any, n (%)	47 (40.9 %)	23 (31.9 %)	24 (55.8 %)	0.023
Sodium, mmol/L, median (IQR)	137.0 (133.0–142.0)	138.0 (133.8–142.0)	137.0 (130.5–140.5)	0.34
Potassium, mmol/L, mean (range)	4.2 (1.9–6.8)	4.1 (1.9–6.8)	4.3 (1.9–6.4)	0.17
Urea, μmol/L, median (IQR)	7.5 (4.3–14.5)	6.7 (4.2–11.6)	10.3 (4.7–15.2)	0.086
Lactate, mmol/L, median (IQR)	2.4 (1.5–4.8)	1.9 (1.3–3.1)	3.9 (2.2–6.8)	<0.001
Creatine, μmol/L, median (IQR)	86.0 (56.0–164.5)	67.5 (52.0–135.2)	112.0 (75.5–180.0)	0.051
WCC, × 10^9^/L, mean (range)	12.5 (0.7–35.5)	12.7 (1.8–35.5)	12.1 (0.7–31.4)	0.68
Bilirubin, μmol/L, median (IQR)	40.0 (16.0–102.0)	28.0 (15.0–82.3)	60.0 (23.0–197.5)	0.027
PT ratio, median (IQR)	1.5 (1.2–2.0)	1.4 (1.2–1.7)	1.9 (1.5–2.2)	<0.001
Albumin, g/L median (IQR)	22.0 (18.0–27.5)	19.0 (16.5–27.5)	21.0 (17.0–27.0)	0.16
Platelets, × 10^9^/L, median (IQR)	120.0 (67.0–215.0)	122.0 (80.0–235.0)	116.0 (46.5–174.0)	0.11
PaO_2_, kPa, median (IQR)	12.1 (9.8–17.5)	12.9 (10.1–19.1)	11.1 (9.7–13.4)	0.11
PaO_2_/FiO_2_ ratio, mean (range)	30.2 (6.0–77.0)	34.0 (7.0–77.0)	23.9 (6.0–59.0)	0.0011
Arterial pH, median (IQR)	7.32 (7.25–7.41)	7.34 (7.26–7.40)	7.31 (7.21–7.42)	0.34
Bicarbonate, mmol/L, mean (range)	20.9 (7.3–35.2)	21.3 (7.7–31.1)	20.2 (7.3–35.2)	0.33
MCV, fL, mean (range)	97.2 (80.0–124.0)	96.4 (80.0–124.0)	98.5 (82.9–119.0)	0.25
Haemocrit, median (IQR)	0.29 (0.24–0.34)	0.29 (0.24–0.34)	0.29 (0.24–0.33)	0.80
APACHE II, mean (range)	16.9 (5.0–29.0)	15.4 (5.0–27.0)	19.4 (9.0–29.0)	<0.001
SOFA, mean (range)	6.4 (0.0–14.0 )	5.4 (0.0–13.0)	8.0 (2.0–14.0)	<0.001
MELD, median (IQR)	18.0 (12.0–24.0)	14.0 (9.8–20.0)	23.0 (17.5–26.0)	<0.001
UKELD, median (IQR)	51.0 (46.0–56.0)	48.0 (44.8–55.3)	54.0 (50.0–59.5)	<0.001
CTP, median (IQR)	10.0 (8.0–11.0)	9.0 (8.0–11.0)	11.0 (9.5–11.0)	<0.001
CTP + L, median (IQR)	13.0 (10.0–16.0)	11.5 (9.0–14.0)	15.0 (13.0–18.0)	<0.001
CLIF-SOFA, median (IQR)	10.0 (7.0–12.0)	9.0 (7.0–12.0)	12.0 (11.0–14.0)	<0.001
RFH, median (IQR)	−0.50 (−3.27–1.34)	−1.60 (−3.78–0.00)	1.29 (−0.27–2.65)	<0.001

*WCC* white cell count, *PT* prothrombin time, *PaO*_*2*_, arterial partial pressure of oxygen, *FiO*_*2*_, inspired oxygen fraction, *MCV* mean corpuscular volume, *APACHE II* acute physiology and chronic health evaluation II, *SOFA* sequential organ failure assessment, *MELD* model of end-stage liver disease, *UKELD* United Kingdom model for end-stage liver disease, *CTP* Child-Turcotte Pugh, *CTP + L* Child-Turcotte Pugh + lactate, *CLIF-SOFA* chronic liver failure sequential organ failure assessment, *RFH* Royal Free Hospital score

The mortality rates in the Glasgow dataset were 30 % for ICU and 46 % for hospital, compared with the London dataset with 37 % ICU and 46 % hospital mortality. Mean APACHE II scores for the Glasgow and London datasets were 23.5 and 16.9, respectively, and the mean SOFA scores were 9.7 and 6.4, respectively.

### Validation of CTP + L

All scoring tools were recalculated for both the Glasgow and London datasets. Within the Glasgow cohort, the RFH scoring tool performed the most accurately (AUC = 0.84), with the CTP + L performing to a similar level (AUC = 0.83). The original CTP score was the least predictive of ICU mortality (AUC = 0.67). CTP + L and RFH scores were the only scores to reach the level of clinical usefulness (AUC > = 0.8) within the Glasgow dataset. Comparison of the AUC of scoring tools applied to each dataset can be seen in Table 4.

**Table 4:** Receiver operating characteristic curve analysis

Scoring tool	AUC	95 % CI	Cut point	Sensitivity	Specificity
Glasgow dataset					
RFH	0.84	0.75–0.93	0.41	0.88	0.67
CTP + L	0.83	0.73–0.93	13.5	0.72	0.84
CLIF-SOFA	0.79	0.69–0.89	11.5	0.72	0.78
MELD	0.77	0.66–0.88	16.5	0.88	0.58
SOFA	0.76	0.65–0.87	9.5	0.80	0.61
APACHE II	0.73	0.61–0.85	25.5	0.60	0.76
UKELD	0.72	0.60–0.84	54.5	0.68	0.81
CTP	0.67	0.55–0.80	10.5	0.52	0.74
London dataset					
RFH	0.77	0.67–0.86	0.14	0.73	0.75
CTP + L	0.75	0.66–0.84	12.5	0.79	0.63
CLIF-SOFA	0.74	0.65–0.84	10.5	0.76	0.67
SOFA	0.71	0.62–0.81	5.5	0.77	0.61
APACHE II	0.71	0.61–0.80	14.5	0.81	0.50
MELD	0.70	0.60–0.80	20.5	0.63	0.76
UKELD	0.69	0.60–0.79	49.5	0.79	0.58
CTP	0.68	0.59–0.78	8.5	0.88	0.47

Cut points and associated sensitivity and specificity were determined by the Youden’s index obtained from receiver operating characteristic curves. *AUC* area under the curve, *APACHE II* acute physiology and chronic health evaluation II, *SOFA* sequential organ failure assessment, *MELD* model of end-stage liver disease, *UKELD* United Kingdom model for end-stage liver disease, *CTP* Child-Turcotte Pugh, *CTP + L* Child-Turcotte Pugh + lactate, *CLIF-SOFA* chronic liver failure sequential organ failure assessment, *RFH* Royal Free Hospital score

On the London dataset the RFH score performed most accurately (AUC = 0.77), with the CTP + L score performing to a similar level (AUC = 0.75). The original CTP score was again the least predictive of ICU mortality (AUC = 0.68). No scoring tool reached the clinically useful AUC of 0.8 in this dataset.

### Binary logistic regression models

As none of the existing scoring tools applied to the London dataset reached the level of clinical usefulness, the raw data were analysed to find the optimum model for predicting ICU outcome. Statistical models were produced by binary logistic regression using the Glasgow dataset, and the optimum model for predicting ICU mortality was determined by ROC curve analysis. The highest AUC from ROC curve analysis in the Glasgow dataset was a model containing lactate, bilirubin, and PaO_2_/FiO_2_ ratio (AUC = 0.89). Model selection using stepwise regression and ANOVA resulted in a model containing lactate, bilirubin, and PaO_2_/FiO_2_ ratio being selected as the optimum (AUC = 0.89). Odds ratios from this model were 1.89 (95 % CI 1.37 − 2.92, *p* = <0.001) for each mmol/L increase of lactate, 1.01 (95 % CI 1.00–1.01, *p* = 0.041) for each μmol/L increase in bilirubin, and 0.96 (95 % CI 0.92–1.00, *p* = 0.062) for each kPa increase in PaO_2_/FiO_2_ ratio for predicting ICU outcome.

A model was produced using the Glasgow data, based on independent predictors of outcome from the London data consisting of PaO_2_/FiO_2_ ratio, PT ratio, and urea, which performed poorly compared to all other models, with an AUC of 0.73. All other models produced using the Glasgow dataset had an AUC >0.8.

The above process was repeated for the London dataset, where models were produced by binary logistic regression and compared using ROC curves. The most predictive model for the London dataset was a combination of PaO_2_/FiO_2_ ratio, PT ratio, and urea obtaining an AUC of 0.78. No model based on the London dataset had an AUC >0.8. Based on stepwise regression analysis and ANOVA, a model containing lactate and PT ratio performed best in the London dataset. Odds ratios for predicting ICU outcome using this model were 1.13 (95 % CI 1.02–1.27, *p* = 0.032) for each mmol/L increase in lactate, and 2.17 (95 % CI 1.20–4.53, *p* = 0.021) for each increment in PT ratio.

A model was produced using the London data, based on the independent predictors of outcome from the Glasgow data, consisting of lactate, bilirubin, and PaO_2_/FiO_2_ ratio. This model performed well compared to all others, with an AUC of 0.76. This is similar to AUC for the RFH and CTP + L scoring tools in this cohort of patients.

### Goodness-of-fit of regression models

In order to determine the usefulness of the statistical models, goodness-of-fit testing was undertaken for both the Glasgow models by applying them to the London dataset, and for the London models by applying them to the Glasgow dataset. The Chi squared goodness-of-fit test was used for all models and phi coefficients compared.

### Collecting encephalopathy grade for the CTP score

When the CTP and CTP + L scores were compared in the Glasgow dataset with and without encephalopathy scores, results showed that there was no statistically significant difference between collecting and not collecting pre-intubation encephalopathy scores, either with the original CTP score (*p* = 0.12) or the modified score CTP + L (*p* = 0.52). Therefore encephalopathy scores do not significantly influence the CTP or CTP + L scores, and it may be unnecessary to collect these.

### Combined performance of the RFH score and CTP + L score

**Fig. 1:**
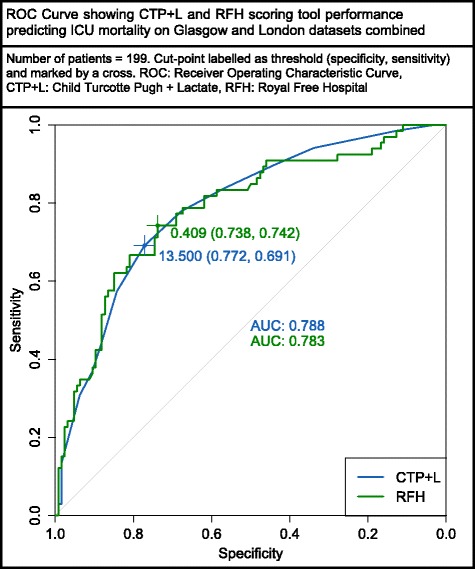
Receiver operating characteristic (*ROC*) curve showing the performance of the Child-Turcotte Pugh plus lactate (CTP + L) and Royal Free Hospital (*RFH*) scoring tools in predicting ICU mortality in the combined Glasgow and London datasets. *AUC* area under the curve

As the RFH score and CTP + L scores were the best performing tools in both datasets, the datasets were combined to create a cohort of 199 patients. ROC curves for the RFH and CTP + L score were produced and are shown in Fig. 1. Both scores performed well in this combined dataset with the CTP + L score having an AUC of 0.79, and the RFH score an AUC of 0.78. There was no statistically significant difference between the ROC curves (*p* = 0.92).

## Discussion

This paper aimed to validate the use of the CTP + L as a method of predicting ICU outcome in patients with cirrhosis admitted to a general ICU. The CTP + L and RFH scoring tools performed similarly in the Glasgow dataset and both performed significantly better than the existing CTP score. When applied to the London dataset to validate the tool, the CTP + L tool performed well but failed to reach the clinically useful AUC of 0.8, which is commonly reported in the literature[10]. The CTP + L tool was more predictive than the CLIF-SOFA score in both datasets.

Mortality rates in the Glasgow and London datasets were similar, with ICU mortality being 30 % and 37 % for the Glasgow and London datasets, respectively, and the hospital mortality the same in both datasets at 46 %. These values are lower than the 48 % and 58 % weighted mean values reported in the literature for ICU and hospital mortality [5]. The SOFA and APACHE II scores were lower in the London dataset than in the Glasgow dataset. These are validated scores for measuring the severity of illness in ICU patients, with higher scores indicating increased severity of illness and greater probability of mortality [22, 23, 29]. The difference in these scores between the two datasets may reflect a difference in admission criteria between the different ICUs. This difference may also reflect the reduced odds ratio associated with lactate in the two datasets, with higher arterial lactate concentration reported within the literature [30, 31] to be associated with higher APACHE II scores and mortality. Future work in this area should explore ICU admission criteria for cirrhosis patients to help understand the care trajectory for this patient cohort.

Although arterial lactate concentration on admission was an independent predictor of ICU mortality in both datasets, the odds ratio for lactate predicting ICU mortality was higher within the Glasgow dataset than the London dataset (odds ratio 1.89 vs 1.13, respectively). Other published papers in this field [16, 17] report odds ratios for lactate higher than that in the London dataset.

Univariate analysis of the Glasgow dataset demonstrated that on admission, lactate, bilirubin, PaO_2_/FiO_2_ ratio, and PT ratio were significant predictors of ICU mortality. These significant predictors were also significant predictors within the London dataset, and other published literature [17]. It was also found in the London dataset that the presence of ascites was a significant predictor of mortality, which was not found in the Glasgow dataset. The significance of lactate, bilirubin and PT ratio as predictors of mortality are logical from a physiological point of view, with elevated lactate demonstrating insufficient oxygen delivery to tissues, or the failure of the liver to metabolise lactate, or both [32]. Elevated bilirubin and PT ratio may represent failure of the liver to metabolise waste products and perform its synthetic function [33, 34].

The RFH score was designed and validated for predicting hospital mortality within a liver transplant centre but not for predicting mortality in a general ICU population [18]. The results of this study validate the use of the RFH score as a prognostic scoring tool for predicting ICU mortality, as demonstrated by its performance in both the Glasgow and London datasets. Although the performance of the RFH is similar to that of the CTP + L score, the RFH is more complex to calculate. Due to the complexity of the calculation, and the requirement to separately calculate the number of failing organ systems, it is clear that this tool is designed for calculation on a computer, which may not be available at the patient’s bedside.

The CTP + L score, however, can be calculated quickly at the patient’s bedside using simple criteria to score 1, 2, or 3 points for each variable, and adding the raw value of the serum arterial lactate to this score, as can be seen in Table 1. CTP + L and RFH scores both predict ICU mortality to a similar degree. However, due to its simplicity, the CTP + L may be a more practical and versatile tool for evaluating patients quickly for admission to the ICU.

From multivariable analysis in the Glasgow dataset, the model that produced the best AUC was that containing lactate, bilirubin and PaO_2_/FiO_2_ ratio (AUC = 0.89). This model also performed well in the London cohort of patients, producing an AUC of 0.76, which is comparable to that of the RFH and CTP + L, as can be seen in Table 4. This same model was selected as the optimum model within the Glasgow dataset based on ANOVA. This suggests that a scoring tool comprising only these factors could be used to predict ICU mortality. This would need to be validated in a larger cohort of patients in order to test its usefulness.

No scoring tool or statistical model from the London dataset reached the clinically useful AUC of 0.8. This is in contrast to the GRI dataset, where all models except one performed with an AUC >0.8. This suggests that patients from within the London dataset have fewer predictive variables compared with the Glasgow dataset, or that the predictive variables within the London cohort were not recorded and therefore not included in this analysis. Goodness-of-fit tests show that although the AUC was lower when the models were applied to the London dataset, the models were still predictive of ICU mortality.

This paper demonstrates that collecting pre-intubation hepatic encephalopathy scores does not increase the predictive values of the CTP or CTP + L scoring tools. Both datasets were combined to compare the performance of the two most predictive scoring tools: the RFH and CTP + L. This ROC curve (Fig. 1) shows that these two tools are similarly matched in predicting ICU outcome in the combined cohort of 199 patients.

### Limitations

Validation of the CTP + L in the London datasets is limited, as the London dataset does not contain pre-intubation encephalopathy score, which is a key component of the CTP + L tool. This lack of pre-intubation encephalopathy score limits the ability to show that encephalopathy score is not required as part of the CTP + L score, and further evidence from another centre with pre-intubation encephalopathy score would be required to prove this conclusively. Both hepatic encephalopathy grade and ascites are subjective in nature and this makes them difficult to assess objectively and apply as part of a scoring tool. Additionally, clinical values for the scoring tools at admission were taken as soon as possible following admission, but in some cases this was delayed by a few hours. This variability in time until first available test results from admission may affect the predictive ability of the scoring tools. The use of first available clinical values does not account for any lead-time bias that may occur. Albumin is routinely administered for patients with cirrhosis as part of current guidelines, however any albumin administered before ICU admission would affect the utility of albumin as a predictive measure of ICU outcome.

## Conclusion

It is known that patients admitted to ICU with cirrhosis have high mortality, however, the mortality rates within these cohorts are more favourable than those published in the literature. The CTP + L and RFH scoring tools are validated prognostic scoring tools for predicting ICU mortality in patients with cirrhotic liver disease admitted to a general ICU department. Collecting hepatic encephalopathy scores is not required for the CTP or CTP + L score, however, this would need to be validated on an external cohort of patients.

## Key messages

- The CTP + L and RFH scoring tools are validated prognostic scoring tools for patients with cirrhosis admitted to a general ICU
- Mortality rates in these cohorts are more favourable than those published in the literature
- Collecting hepatic encephalopathy scores may not be necessary for the CTP or CTP + L score in patients admitted to the general ICU, although this requires external validation

## Abbreviations

ANOVA: analysis of variance
APACHE II: acute physiology and chronic health evaluation II
AUC: area under the curve
CLIF-SOFA: chronic liver failure-sequential organ failure assessment score
CTP: Child-Turcotte Pugh
FiO_2_: inspired oxygen fraction
GAHS: Glasgow alcoholic hepatitis score
GCS: Glasgow coma scale
GRI: Glasgow Royal Infirmary
MAP: mean arterial blood pressure
MELD: model of end-stage liver disease
PaCO_2_: arterial partial pressure of carbon dioxide
PaO_2_: arterial partial pressure of oxygen
PT: prothrombin time
RFH: Royal Free Hospital
ROC: receiver operating characteristic
SIMD: Scottish Index of Multiple Deprivation
SOFA: sequential organ failure assessment
UKELD: United Kingdom model for end-stage liver disease
